# Neural Mechanisms Underlying Conscious and Unconscious Gaze-Triggered Attentional Orienting in Autism Spectrum Disorder

**DOI:** 10.3389/fnhum.2017.00339

**Published:** 2017-06-28

**Authors:** Wataru Sato, Takanori Kochiyama, Shota Uono, Sayaka Yoshimura, Motomi Toichi

**Affiliations:** ^1^Department of Neurodevelopmental Psychiatry, Habilitation and Rehabilitation, Graduate School of Medicine, Kyoto UniversityKyoto, Japan; ^2^Brain Activity Imaging Center, Advanced Telecommunications Research Institute InternationalKyoto, Japan; ^3^Faculty of Human Health Science, Graduate School of Medicine, Kyoto UniversityKyoto, Japan; ^4^The Organization for Promoting Neurodevelopmental Disorder ResearchKyoto, Japan

**Keywords:** amygdala, attentional orienting, autism spectrum disorder (ASD), eye gaze, functional magnetic resonance imaging (fMRI), subliminal presentation

## Abstract

Impaired joint attention represents the core clinical feature of autism spectrum disorder (ASD). Behavioral studies have suggested that gaze-triggered attentional orienting is intact in response to supraliminally presented eyes but impaired in response to subliminally presented eyes in individuals with ASD. However, the neural mechanisms underlying conscious and unconscious gaze-triggered attentional orienting remain unclear. We investigated this issue in ASD and typically developing (TD) individuals using event-related functional magnetic resonance imaging. The participants viewed cue stimuli of averted or straight eye gaze direction presented either supraliminally or subliminally and then localized a target. Reaction times were shorter when eye-gaze cues were directionally valid compared with when they were neutral under the supraliminal condition in both groups; the same pattern was found in the TD group but not the ASD group under the subliminal condition. The temporo–parieto–frontal regions showed stronger activation in response to averted eyes than to straight eyes in both groups under the supraliminal condition. The left amygdala was more activated while viewing averted vs. straight eyes in the TD group than in the ASD group under the subliminal condition. These findings provide an explanation for the neural mechanisms underlying the impairment in unconscious but not conscious gaze-triggered attentional orienting in individuals with ASD and suggest possible neurological and behavioral interventions to facilitate their joint attention behaviors.

## Introduction

Individuals with autism spectrum disorder (ASD) are characterized by qualitative impairments in social interaction (American Psychiatric Association, 2013). One of the earliest developmental features of these social impairments is abnormal joint attention (Mundy et al., 1994). For example, when an adult suddenly turns his/her eye gaze toward an object during an interaction, children with ASD are less likely to follow the gaze than are typically developing (TD) children (Leekam et al., 1997).

Experimental behavioral studies have provided information regarding typical and atypical components in the reflexive joint attention of individuals with ASD. By presenting eye-gaze cues supraliminally using Posner’s cueing paradigm (Posner, 1980), several studies reported that the ability to orient attention in response to another’s eyes is comparable in TD and ASD individuals (Chawarska et al., 2003; Okada et al., 2003; Swettenham et al., 2003; Kylliäinen and Hietanen, 2004; Senju et al., 2004; Johnson et al., 2005; Vlamings et al., 2005; Nation and Penny, 2008; Rutherford and Krysko, 2008; Pruett et al., 2011; Landry and Parker, 2013; Kirchgessner et al., 2015), although impairments in orienting were also reported (Ristic et al., 2005; Goldberg et al., 2008). For example, Kylliäinen and Hietanen (2004) presented face stimuli with the eyes showing either an averted gaze or a straight gaze, and then a target was presented on the right or left side of the face. The reaction time (RT) for detecting the target was shorter after a face with a valid averted gaze (i.e., directionally congruent with target location) than a face with straight eyes or an invalid averted gaze in both ASD and TD participants. In contrast, another previous study (Sato et al., 2010) presented subliminal eye-gaze cues using a cueing paradigm and reported that the cueing effect was evident in the TD group, which is similar to previous studies (Sato et al., 2007; Xu et al., 2011; Al-Janabi and Finkbeiner, 2012; Bailey et al., 2014), but not in the ASD group. Taken together, even though findings under the supraliminal condition are inconsistent and those under the subliminal condition are scarce, these data suggest that gaze-triggered attentional orienting is intact in response to supraliminally presented eyes but impaired in response to subliminally presented eyes in individuals with ASD.

Despite accumulating behavioral data, the neural mechanisms underlying typical conscious and atypical unconscious gaze-triggered attentional orienting in individuals with ASD remain unclear. Several functional magnetic resonance imaging (fMRI) studies have investigated the conscious component in TD participants by measuring brain activation in response to averted vs. straight eyes that were supraliminally presented in the framework of the cueing paradigm (Kingstone et al., 2004; Hietanen et al., 2006; Tipper et al., 2008; Greene et al., 2009; Sato et al., 2009, 2016a; Engell et al., 2010; Cazzato et al., 2012; Callejas et al., 2013). The comparison between averted and straight eye gaze allows for investigation of the neural correlates underlying attentional orienting triggered by eye gaze, while controlling for basic visual processes and eye processes *per se*. Some studies have consistently reported that the attentional orienting triggered by eye gaze is associated with activation in the temporo–parieto–frontal regions (Tipper et al., 2008; Greene et al., 2009; Sato et al., 2009, 2016a), which constitutes the attentional neural network (Corbetta and Shulman, 2002; Grosbras et al., 2005). In contrast, only one previous neuroimaging study investigated the neural mechanisms underlying conscious gaze-triggered attentional orienting in individuals with ASD using the cueing paradigm (Greene et al., 2011). The researchers found less activation in some brain regions, including the middle temporal gyrus and anterior cingulate cortex, during attentional orienting elicited by eye-gaze cues in the ASD group than in the TD group. These data suggest that there is reduced activation in the cortical attentional network in response to eye gaze in individuals with ASD. At the same time, both the TD and ASD groups showed activation of the parietal cortices in response to eye gaze, suggesting some commonalities in attentional network activation across groups. However, statistical analyses were not conducted to identify areas of common activation across the ASD and TD groups. Although other studies investigated gaze processing in ASD and TD groups using different paradigms, they also did not test the commonalities across the groups (Dichter and Belger, 2007; Vaidya et al., 2011). Based on these data, together with the aforementioned behavioral data showing comparable amounts of conscious gaze-triggered attentional orienting in TD and ASD groups, we hypothesized that several similarities and differences would be demonstrated by TD and ASD groups in the activation of the temporo–parieto–frontal attentional network in response to supraliminally presented averted vs. straight eyes.

Furthermore, no study to date has investigated the neural mechanisms underlying unconscious gaze-triggered attentional orienting in individuals with ASD. A recent neuroimaging study of TD participants showed that subliminally presented eye gaze activated subcortical structures, including the amygdala, in addition to the cortical attentional network (Sato et al., 2016a). Another neuroimaging study reported that amygdala activity of a cortical blindness patient changed depending on the direction of unseen eyes (Burra et al., 2013). An intracranial field potential recording study showed that amygdala activity in response to eyes was rapid, indicating that it can occur prior to conscious awareness (Sato et al., 2011, 2013). These data indicate that the amygdala may be involved in the eye-related unconscious processing of TD participants. Several previous neuroimaging studies in individuals with ASD reported that the amygdala showed weakened activation in response to averted gaze (Zürcher et al., 2013) as well as other facial information, such as emotional facial expressions (Baron-Cohen et al., 1999; Critchley et al., 2000; Ashwin et al., 2007; Sato et al., 2012). Several anatomical studies also reported structural abnormalities, such as reduced numbers of neurons (Schumann and Amaral, 2006) and reduced gray matter volumes (Nacewicz et al., 2006; Via et al., 2011), in the amygdala of ASD individuals. Based on these data, together with the aforementioned behavioral findings showing impaired unconscious gaze-triggered attentional orienting in individuals with ASD, we hypothesized that there would be lower levels of activation in the amygdala in response to subliminally presented averted vs. straight eyes in the ASD group compared with the TD group.

We tested these hypotheses in a TD group and an ASD group by measuring brain activity using rapid event-related fMRI. To reduce the effects of confounding factors, such as difficulties understanding task instructions and motor control issues, the present study recruited high-functioning adults with ASD. The participants viewed cue stimuli consisting of averted or straight eyes presented supraliminally or subliminally and were subsequently required to localize a target. Cognitive conjunction analyses (Price and Friston, 1997) were performed to identify brain regions that were commonly active in response to averted vs. straight eyes in the TD and ASD groups under each presentation condition. Additionally, the interactions between group and gaze direction were analyzed to determine the brain regions that showed different activation for averted vs. straight eyes across groups under each presentation condition.

## Materials and Methods

### Participants

The ASD group included 16 adults (one female and 15 males; mean ± *SD* [range] age, 26.1 ± 6.3 [19–42] years); seven were diagnosed with Asperger’s disorder and nine were diagnosed with pervasive developmental disorder not otherwise specified, who exhibited mild Asperger’s disorder symptoms. These diagnoses were made based on the Diagnostic and Statistical Manual of Mental Disorders (DSM), 4th Edition, Text Revision (American Psychiatric Association, 2000); in the DSM-5 (American Psychiatric Association, 2013), both of these diagnoses are included within the ASD category. The diagnosis was made based on a stringent procedure in which every item of the ASD diagnostic criteria was assessed in interviews with the participants and their parents (and professionals who helped them, if any) conducted by two psychiatrists with expertise in developmental disorders. Individuals with neurological and psychiatric problems other than those associated with ASD were excluded. No participant was taking medication. The full-scale intelligence quotient scores of participants were assessed using the Wechsler Adult Intelligence Scale-Revised (Nihon Bunka Kagakusha, Tokyo, Japan) and were in the normal range (mean ± *SD* [range], 114.2 ± 12.4 [97–132]). The severity levels of the symptoms in the participants were quantitatively assessed using the Childhood Autism Rating Scale (Schopler et al., 1986); the scores (mean ± *SD* [range], 23.2 ± 3.4 [18.0–29.5]) were comparable to those from previous studies that included high-functioning individuals with ASD (Koyama et al., 2007; Uono et al., 2011; Sato et al., 2012; *t*-test, *p* > 0.10).

The control group included 17 adults (two females and 15 males; mean ± *SD* [range] age, 24.0 ± 4.5 [19–39] years); the control participants had no neurological or psychiatric problems and were matched with the ASD group for age (*t*-test, *p* > 0.10) and sex (*χ*^2^-test, *p* > 0.10). The data of some participants in the control group were reported as part of a previous study (Sato et al., 2016a).

All participants had normal or corrected-to-normal visual acuity and were right handed, as assessed by the Edinburgh Handedness Inventory (Oldfield, 1971). After the experimental procedures had been fully explained, written informed consent was obtained from all participants. This study was approved by the Ethics Committee of the Primate Research Institute, Kyoto University, and was conducted in accordance with the approved guidelines.

### Stimuli

The eye-gaze stimuli were almost identical to those used in a previous study (Uono et al., 2009). Photographs of two models (one female and one male) showing a neutral facial expression were selected from a standard set (Ekman and Friesen, 1976). To manipulate gaze direction, the irises and pupils of the eyes were extracted from the original photographs and inserted at the right or left side of the eyeball using Photoshop 5.0 (Adobe, San Jose, CA, USA). We cropped the photographs in an elliptical shape, 2.7° wide and 3.8° high, to exclude hair and background.

A mosaic image was created from a neutral facial expression by dividing the photos into a 50 × 40 grid and randomly rearranging the pieces, rendering the resulting photograph unrecognizable as a face. The letter “T” (0.6° wide × 0.6° high), presented 5.7° to the left or right of the center of the screen, was used as a target stimulus.

### Apparatus

The experiments were controlled using Presentation 10.0 (Neurobehavioral Systems, Albany, CA, USA). The stimuli were projected from a liquid crystal projector (DLA-G150CL; Victor Electronics, Brussels, Belgium) at a refresh rate of 75 Hz to a mirror positioned in front of the participants. Responses were obtained using a response box (Response Pad; Current Designs, Philadelphia, PA, USA).

### Procedure

The participants completed a total of 240 trials presented in two runs of 120 trials. Each run lasted 427.5 s and corresponded to one of the presentation conditions (supraliminal and subliminal); the order of the conditions was counterbalanced across participants. Each run consisted of an equal number of trials for the gaze-direction conditions (i.e., 40 trials each for averted-left, averted-right and straight eye gaze) and cue-validity conditions (i.e., 40 trials each for valid, neutral and invalid); equal numbers of trials for the gaze-direction and cue-validity conditions have been used in several previous behavioral studies (e.g., Friesen and Kingstone, 1998). The order and temporal patterns of these conditions were determined through simulations (see Dale, 1999; Friston et al., 1999; Morita et al., 2008). The efficiency with which differential activation for averted and straight eyes was detected was maximized while also maximizing the efficiency with which the evoked response under each condition was estimated. Accordingly, null events were included at a probability of 25% and the inter-trial intervals varied among 2500, 5000, 7500, 10,000 and 12,500 ms. A short break was interposed after the first run and 10 practice trials preceded the experimental trials.

For each trial, a fixation point (i.e., a small white “+”) was presented for 500 ms at the center of the screen. The gaze cue was then presented at the same location. Under the supraliminal condition, the gaze cue was presented for 200 ms and no masking followed. Under the subliminal condition, the gaze cue was presented for 13 ms and was followed by the presentation of the mask in the same location for 187 ms. Then, a target was presented in either the left or right peripheral visual field (5.0° from the center) 100 ms after the gaze cue disappeared under the supraliminal condition or the mask disappeared under the subliminal condition. The target remained until a response was made or 1700 ms elapsed. As in previous studies (e.g., Friesen and Kingstone, 1998; Sato et al., 2007), participants were instructed to localize targets as quickly as possible by pressing buttons using their left or right index finger. Participants were told that the stimuli preceding the targets did not predict anything.

Following image acquisition, the subjective thresholds of the participants were assessed to ensure subliminal presentations. A total of 30 trials were performed; 24 were similar to the trials under the subliminal condition during image acquisition, except that the gaze cues were presented for 13, 27, 40 and 53 ms in each of six trials. We also included six trials with no gaze cue as the baseline condition. The order of trials was randomized. Participants were asked, “Did you see the gaze? If so, report the gaze direction.” Participants responded either “Yes” or “No”; when the response was “Yes”, they reported the gaze direction.

### Image Acquisition

Image scanning was performed on a 3-T scanning system (MAGNETOM Trio, A Tim System; Siemens, Malvern, PA, USA) using a 12-channel head coil. The head position was fixed by lateral foam pads. The functional images consisted of 40 consecutive slices parallel to the anterior–posterior commissure plane, and covered the whole brain. A T2*-weighted gradient-echo echo-planar imaging sequence was used with the following parameters: repetition time (TR) = 2500; echo time (TE) = 30 ms; flip angle = 90°; matrix size = 64 × 64; voxel size = 3 × 3 × 4 mm. After the acquisition of functional images, a T1-weighted high-resolution anatomical image was obtained using a magnetization-prepared rapid-acquisition gradient-echo sequence (TR = 2250 ms; TE = 3.06 ms; flip angle = 9°; field of view = 256 × 256 mm; voxel size = 1 × 1 × 1 mm).

### Behavioral Data Analysis

Behavioral data were analyzed using SPSS 16.0J (SPSS Japan, Tokyo, Japan). The mean RT of correct responses was calculated for each condition for each participant, excluding measurements beyond the total mean ± 3 *SD*, which were considered artifacts (mean% ± *SD* [range], 3.5 ± 3.4 [0.8–15.0] and 2.8 ± 1.8 [0.0–7.5] for the TD and ASD groups, respectively; *t*-test, *p* > 0.10). The RTs after log transformation were analyzed using a two-way analysis of variance (ANOVA) with group (TD and ASD) and cue validity (neutral, valid and invalid) as factors. Significant interactions were analyzed further with follow-up tests for simple main effects (see Kirk, 1995). Even when the interactions were not significant, *t-tests* for each group under each presentation condition were conducted to investigate differences between the valid and neutral conditions, which was the effect of primary interest in the present study. Although no specific predictions were made, the differences between the invalid and neutral conditions were also explored. Note that several previous studies investigating attention orienting triggered by eye-gaze reported no evident differences between invalid and neutral conditions in either the TD or ASD groups (specifically with short cue–target duration, as in this study; e.g., Friesen and Kingstone, 1998; Kylliäinen and Hietanen, 2004). Because preliminary analyses showed small error rates for the TD and ASD groups (mean% ± *SD* [range], 0.3 ± 0.4 [0.0–1.3] and 1.1 ± 1.0 [0.0–3.8], respectively; *t-test*, *p* > 0.10) and no evidence of a speed–accuracy trade-off, only the RT results are reported. The preliminary analyses also showed that the effects of group and cue validity were remained when possible confounding factors (sex and age) were accounted for as covariates; to simplify the model and due to an insufficient design for investigating these factors (see “Discussion” Section), they were not included in the reported analyses.

For the threshold data, the percentages of “Yes” responses under the no-gaze vs. under the 13-ms conditions were compared using a paired *t*-test in each group. The correct response percentages under the 13-ms conditions were also compared to chance levels using a one-sample *t*-test in each group. The group differences for the “Yes” and correct responses were further analyzed using two-way ANOVAs with group (TD and ASD) and presentation duration of two-levels (no gaze and 13 ms for “Yes” responses) or all-levels (no gaze, 13 ms, 27 ms, 40 ms, or 53 ms for “Yes” responses; 13 ms, 27 ms, 40 ms and 53 ms for correct responses) as factors.

All test results were considered to indicate statistical significance at *p* < 0.05.

### Image Analysis

Image analyses were performed using the statistical parametric mapping package SPM12^1^, implemented in MATLAB R2009a (MathWorks, Natick, MA, USA). First, to correct for head motion, functional images of each run were realigned using the first scan as a reference. The realignment parameters revealed only a small motion correction for both the TD and ASD groups (mean ± *SD* [range] maximum *x*/*y*/*z* translation [mm], 0.58 ± 0.31 [0.09–1.10] and 0.64 ± 0.43 [0.08–1.32]; mean ± *SD* [range] maximum pitch/roll/yaw rotation [°], 0.01 ± 0.01 [0.00–0.02] and 0.01 ± 0.01 [0.00–0.03], respectively; *t*-test, *p* > 0.10). Next, all functional images were corrected for slice timing. Then, the T1 anatomical image was coregistered to the mean of the functional images. Subsequently, all anatomical and functional images were normalized to the Montreal Neurological Institute space using the anatomical image-based unified segmentation-spatial normalization approach (Ashburner and Friston, 2005). Finally, the spatially normalized functional images were resampled to a voxel size of 2 × 2 × 2 and smoothed with an isotopic Gaussian kernel of 8-mm full-width at half-maximum to compensate for anatomical variability among participants.

We used random-effects analyses to identify significantly activated voxels at the population level (Holmes and Friston, 1998). First, a single-subject analysis was performed (Friston et al., 1995). The task-related regressor for each condition (averted-left, averted-right and straight eye-gaze) was modeled by a series of delta functions convolving it with a canonical hemodynamic response function for each presentation condition in each participant. Because the primary focus of the present study was brain activation in response to stimuli, trials in which participants made errors or artifacts were present during the behavioral responses were not excluded. The realignment parameters were used as covariates to account for motion-related activation as covariates. We used a high-pass filter with a cutoff period of 128 to eliminate the artifactual low-frequency trend. To correct the global fluctuation related to motion artifacts, global scaling was conducted. Serial autocorrelation was accounted for using a first-order autoregressive model. The contrast images of each presentation condition were entered into a two-way ANOVA model with group (TD and ASD) and direction (averted and straight) as factors for the second-level random-effects analysis. Because the preliminary analyses revealed that the effects of group and cue validity remained when sex and age were accounted for as covariates, these factors were not included in the analyses.

To test for commonalities across groups in brain activity in response to averted vs. straight eyes under each presentation condition, a conjunction analysis was performed using interaction masking (Price and Friston, 1997), as in a previous study (Sato et al., 2009). For this analysis, a main-effect analysis of direction (averted vs. straight) was conducted using *T*-statistics. To search for brain areas that showed common activity across groups, the main effect was exclusively masked by *F*-tests of group × direction interactions at a threshold of *p* < 0.05 (uncorrected). Voxels were identified as significantly activated if they reached the height threshold of *p* < 0.01 (uncorrected) with the extent threshold of 100 contiguous voxels, which roughly corresponded to *p* < 0.05 (corrected) determined by Monte Carlo simulations (Ramasubbu et al., 2014), to produce the best balance between Type I and Type II errors (Lieberman and Cunningham, 2009).

To test for differences across groups in brain activity in response to averted vs. straight eyes under each presentation condition, interactions between group (TD vs. ASD) and direction (averted vs. straight) were analyzed using *T*-statistics. Thresholds were identical to those used in the aforementioned commonality analysis. Additionally, the different types of interactions (e.g., group [ASD vs. TD] and direction [averted vs. straight]) were investigated for descriptive purposes.

Brain structures other than the amygdala were labeled anatomically and identified according to Brodmann’s areas using the Automated Anatomical Labeling atlas (Tzourio-Mazoyer et al., 2002) and Brodmann Maps^2^, respectively, with MRIcron^3^. The amygdala, as well as its subregions, were identified based on a cytoarchitectonic map derived from human postmortem brain data using Anatomy Toolbox 2.0 (Amunts et al., 2005; Eickhoff et al., 2005).

## Results

### Threshold Assessment

The mean ± *SE* percentages of “Yes” (seen) responses and correct responses are presented in Table 1. Significant differences in “Yes” responses under the 13 ms and no gaze conditions were not observed in either the TD or the ASD group (*t* < 0.72, *p* > 0.10). The correct responses under the 13 ms condition were also significantly lower than chance in both groups (*t* > 8.94, *p* < 0.001). The results confirmed that the subliminal cues under the current (i.e., 13 ms) condition did not elicit conscious awareness in either group. Two-way ANOVAs with group and presentation duration (two-levels of no gaze and 13 ms conditions or all levels) as factors revealed no significant group-related main effects or interactions for either “Yes” responses or correct responses (*F* < 0.99, *p* > 0.10), which indicated that there were comparable patterns across groups.

**Table 1:** Mean (with *SE*) % responses for threshold assessment.

Response	Group	No gaze		13 ms		27 ms		40 ms		53 ms
%Yes (seen)	TD	4.9	(3.2)	5.9	(3.2)	3.9	(2.7)	16.7	(5.5)	33.3	(8.1)
	ASD	4.2	(2.8)	8.3	(5.5)	13.5	(6.3)	19.8	(7.5)	37.5	(10.0)
%Correct	TD	-	-	2.9	(1.6)	2.9	(2.1)	8.8	(3.5)	22.5	(5.7)
	ASD	-	-	4.2	(3.2)	6.3	(3.7)	11.5	(5.4)	26.0	(7.8)

*TD, typically developing; ASD, autism spectrum disorder*.

### RT

**Figure 1:**
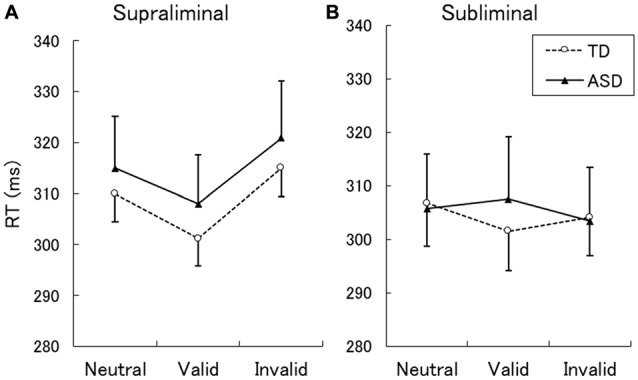
Mean (with *SE*) reaction time (RT) under the supraliminal **(A)** and subliminal **(B)** presentation conditions. TD, typically developing; ASD, autism spectrum disorder.

The mean ± *SE* RTs for correct responses are shown in Figure 1. A two-way ANOVA with group and cue validity as factors for the supraliminal condition revealed a significant main effect of only cue validity (*F*_(2,62)_ = 17.86, *p* < 0.001). The main effect of group and the interaction between group and cue validity were not significant (*F* < 0.17, *p* > 0.1). For purposes of confirmation, we conducted a series of *t*-tests and confirmed that RTs for valid cues were shorter than those for neutral and invalid cues in both the TD and ASD groups (*t* > 2.15, *p* < 0.05). The differences between RTs for the neutral and invalid cues were only marginally significant in the TD group (*t*_(16)_ = 1.90, *p* < 0.1) and were not significant in the ASD group (*t*_(15)_ = 1.64, *p* > 0.1).

A two-way ANOVA for the subliminal condition revealed a significant interaction only between group and cue validity (*F*_(2,62)_ = 3.74, *p* < 0.05). The main effects of group and cue validity were not significant (*F* < 1.75, *p* > 0.1). Follow-up analyses of the interaction indicated that the simple main effect of cue validity was significant in the TD (*F*_(2,62)_ = 3.61, *p* < 0.05) but not in the ASD group (*F*_(2,62)_ = 1.87, *p* > 0.1). In the TD group, a significant difference was found between the valid and neutral conditions (*t*_(62)_ = 2.72, *p* < 0.01), which indicated that the RTs for the valid cues were shorter than those for neutral cues. The differences between the valid and invalid conditions and between the neutral and invalid conditions were not significant (*t* < 1.46, *p* > 0.1). To confirm these findings, a series of *t*-tests on the data from the ASD group was conducted and no significant differences were identified among the cue-validity conditions (*t* < 1.57, *p* > 0.1).

### Commonalities in Neural Activity

**Figure 2:**
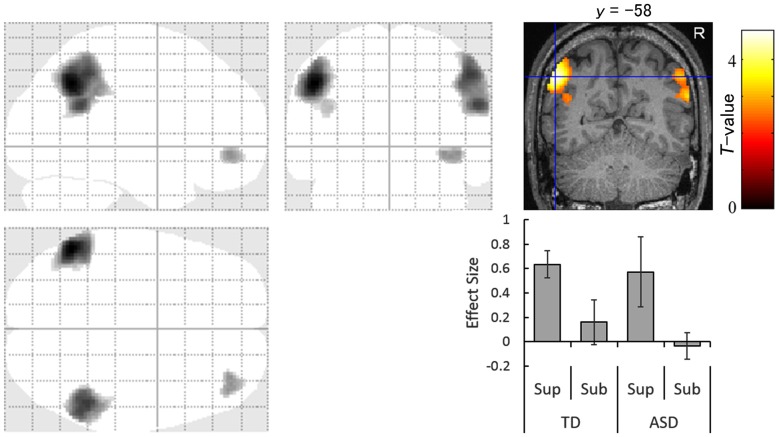
Statistical parametric maps indicating regions that were significantly more activated in both typically developing (TD) and autism spectrum disorder (ASD) groups in response to averted than to straight eyes under the supraliminal (Sup) presentation condition. Areas of activation are rendered on the glass brain (left) and the brain of a representative participant (upper right). The blue cross indicates the activation focus at the left angular gyrus (*x* = −52, *y* = −58, *z* = 40) and the red–yellow color scale represents the *T*-value. Effect size indicates mean (with *SE*) beta value differences between averted eyes and straight eyes (lower right). R, right hemisphere; Sub, subliminal.

The images under each presentation condition were analyzed using a two-way ANOVA model with group and direction as factors. The conjunction analysis testing the commonality across groups for the main effect of direction with an exclusive mask of the group × direction interaction for the supraliminal condition revealed that averted eyes activated the bilateral inferior parietal lobules, which covered parts of the posterior superior temporal gyri, and the right inferior frontal gyrus significantly more than the straight eyes in both the TD and ASD groups (Table 2; Figure 2).

**Table 2:** Brain regions exhibiting significant activation in both the typically developing and autism spectrum disorder groups in response to averted eyes vs. straight eyes under each presentation condition.

Presentation	Side	Area	Region	BA	Coordinates			*Z*-value	Cluster size (mm^3^)
					*x*	*y*	*z*		
Supraliminal	L	Parietal	Angular gyrus	39	−52	−58	40	4.62	7880
	R	Parietal	Inferior parietal lobule	40	50	−48	46	3.97	9048
	R	Parietal	Angular gyrus	22	56	−52	26	3.77	
	R	Frontal	Inferior frontal gyrus	47	34	46	−6	3.14	1160
Subliminal				None

*BA, Brodmann’s area; L, left; R, right*.

The conjunction analysis for the subliminal condition did not reveal any significant activation.

### Differences in Neural Activity

**Figure 3:**
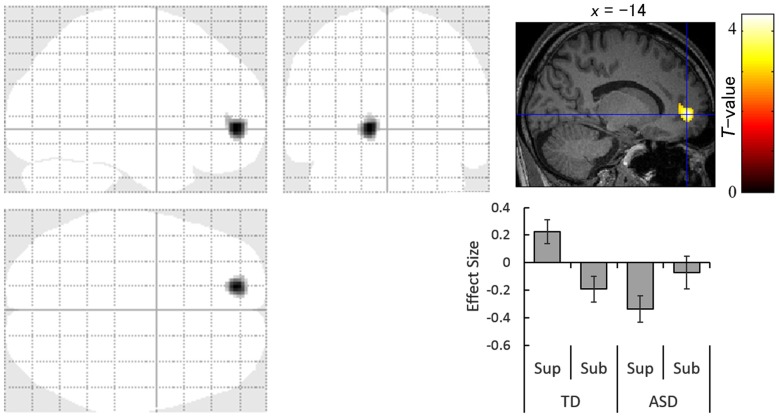
Statistical parametric maps indicating regions with significantly more activation in the typically developing (TD) group than in the autism spectrum disorder (ASD) group in response to averted than to straight eyes under the supraliminal (Sup) presentation condition. Areas of activation are rendered on the glass brain (left) and the brain of a representative participant (upper right). The blue cross indicates the activation focus at the left anterior cingulate gyrus (*x* = −14, *y* = 50, *z* = −2), and the red–yellow color scale represents the *T*-value. Effect size indicates mean (with *SE*) beta value differences between averted vs. straight eyes (upper right). Sub, subliminal.

The contrast of the interaction between group (TD vs. ASD) and direction (averted vs. straight) for the supraliminal condition revealed significantly more activation in the left anterior cingulate gyrus in response to averted eyes vs. straight eyes in the TD group compared with the ASD group (Table 3; Figure 3).

**Table 3:** Brain regions exhibiting significant interactions between group (typically developing > autism spectrum disorder) and direction (averted eyes > straight eyes) under each presentation condition.

Presentation	Side	Area	Region	BA	Coordinates			*Z*-value	Cluster size (mm^3^)
					*x*	*y*	*z*			
Supraliminal	L	Frontal	Anterior cingulate gyrus	10	−14	50	−2	4.30	1344
Subliminal	L	Temporal	Middle temporal gyrus	20	−40	6	−28	3.45	984
	L	Subcortex	Amygdala	-	−32	−6	−24	2.86	
	L	Occipital	Cuneus	23	−8	−64	22	3.21	1656

*BA, Brodmann’s area; L, left*.

**Figure 4:**
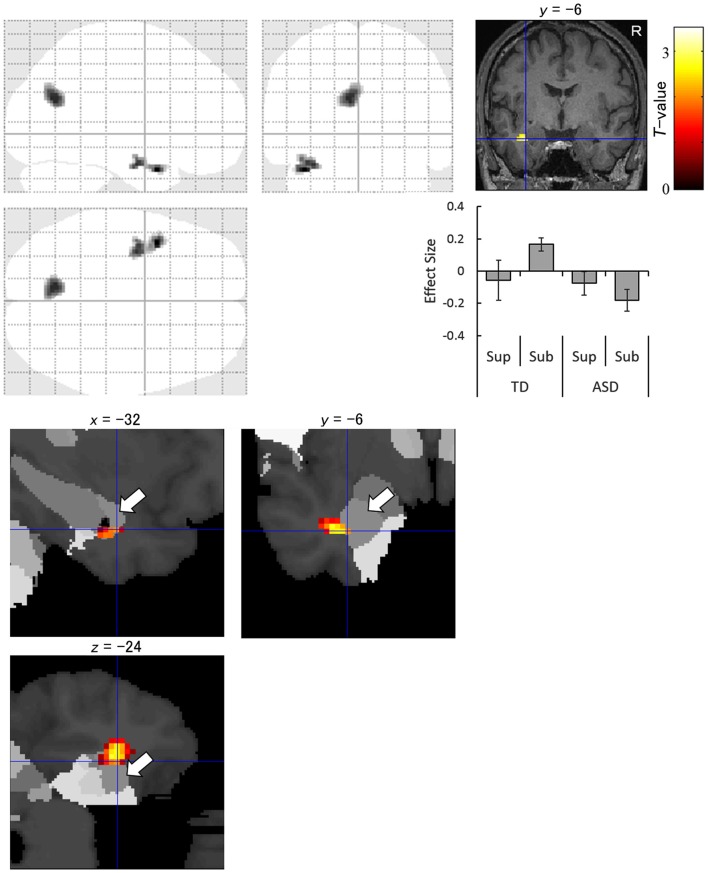
Statistical parametric maps indicating regions with significantly more activation in the typically developing (TD) group than in the autism spectrum disorder (ASD) group in response to averted than to straight eyes under the subliminal (Sub) presentation condition. Areas of activation are rendered on the glass brain (upper left), the brain of a representative participant (upper right), and the cytoarchitectonic map derived from human postmortem brain data (lower; white arrows indicate the amygdala). Blue crosses indicate the activation focus at the left amygdala (*x* = −32, *y* = −6, *z* = −24), and the red–yellow color scale represents the *T*-value. Effect size indicates mean (with *SE*) beta value differences between averted eyes and straight eyes (middle right). R, right hemisphere; Sup, supraliminal.

The interaction analysis for the subliminal condition showed significantly stronger activation in response to averted eyes vs. straight eyes in the anterior temporal lobe, including the amygdala, and in the cuneus in the left hemisphere in the TD group than in the ASD group (Figure 4). The amygdala activation was validated using the cytoarchitectonic map (Amunts et al., 2005; Eickhoff et al., 2005), which indicated that the activation cluster covered the amygdala and that the peak was located in the amygdala laterobasal subregion with a 70% probability (Figure 4).

The contrasts of the other interactions under the supraliminal and subliminal conditions did not exhibit any significant activation.

## Discussion

The present behavioral results showed that, under the supraliminal condition, valid cues shortened RTs more than did neutral cues for both TD and ASD participants. These findings suggest that normal attentional orienting is triggered by supraliminally presented eyes in both the TD and ASD groups, which is consistent with several previous studies of TD and ASD individuals (e.g., Kylliäinen and Hietanen, 2004). The neutral and invalid conditions did not significantly differ in either group, which is also consistent with previous findings (e.g., Friesen and Kingstone, 1998; Kylliäinen and Hietanen, 2004). This lack of delay for invalid cues is thought to be a unique characteristic related to reflexive attention orienting (Friesen and Kingstone, 1998). Under the subliminal condition, the same cueing effect for valid vs. neutral cues was found in the TD group but not in the ASD group. The present results provide evidence that attentional orienting triggered by subliminally presented eyes occurs in TD individuals but is impaired in ASD individuals, which is in line with the findings of a previous study (Sato et al., 2010). The differences between the valid and invalid conditions did not reach significance in the present study, which differs from previous findings (Sato et al., 2010); however, this discrepancy may be accounted for by methodological differences across studies. For example, the presentation periods of the eye-gaze stimuli were shorter and there were fewer trials in the present study. Taken together, these behavioral data suggest that individuals with ASD exhibit intact attention orienting in response to supraliminally presented eyes but impaired attention orienting to subliminally presented eyes.

Our conjunction analysis of fMRI data under the supraliminal condition revealed that the bilateral temporo–parietal regions, covering the inferior parietal lobule and posterior superior temporal gyrus, and the right inferior frontal gyrus were more activated in response to averted eyes than straight eyes in both the TD and ASD groups. The activation of these regions in association with attentional orienting triggered by averted eyes among TD participants is consistent with the results of several previous studies (Tipper et al., 2008; Greene et al., 2009; Sato et al., 2009, 2016a). The activation of some parietal regions in both groups is also consistent with a previous study (Greene et al., 2011). However, that study did not statistically test the commonalities in neural activities across the TD and ASD groups. Thus, the present results extend these previous findings and indicate that the temporo–parieto–frontal attentional network is commonly activated for conscious gaze-triggered attentional orienting in TD and ASD individuals.

Simultaneously, our interaction analysis of the supraliminal fMRI data revealed that the left anterior cingulate gyrus was less activated in the ASD group than in the TD group during the attentional orienting triggered by supraliminally presented eye gaze. This result is consistent with a previous neuroimaging study reporting that some regions in the attentional network, including the anterior cingulate gyrus, were less activated in the ASD than in the TD group in response to averted eyes (Greene et al., 2011). These data indicate that the neural mechanisms for the conscious attentional orienting triggered by gaze may differ, in part, between ASD and TD groups despite their similarity in behavioral patterns.

The fMRI data interaction analysis for the subliminal condition revealed that the left anterior temporal region, including the amygdala, was less activated in the ASD group compared with the TD group during the attentional orienting triggered by subliminally presented eye gaze. The reduced activation of the amygdala in the ASD group for averted vs. straight eyes, even stronger activation for straight vs. averted eyes, is consistent with the results of a previous neuroimaging study that tested brain activation in response to averted vs. straight eye gaze in supraliminally presented fearful expressions (Zürcher et al., 2013). Several neuroimaging studies also reported less activation in the amygdala in ASD participants during the observation of emotional facial expressions (Baron-Cohen et al., 1999; Ashwin et al., 2007; Sato et al., 2012). In the present study, the focus of activation was localized in the laterobasal subregion of the amygdala. Consistent with this result, a previous anatomical study has reported that individuals with ASD have a reduced number of neurons in this amygdala subregion compared with TD individuals (Schumann and Amaral, 2006). This subregion is also involved in social functions in monkeys (Nakamura et al., 1992; Gothard et al., 2007) and humans (Hurlemann et al., 2008; Sato et al., 2016b). Furthermore, electrophysiological studies in monkeys demonstrated that the amygdala, specifically the laterobasal subregion, is related to attention orienting triggered by biologically significant stimuli (Peck et al., 2013; Peck and Salzman, 2014). The reduced activation in response to subliminally-presented averted eyes compared with straight eyes in the ASD group was also observed in the occipital cortex. The activation of the occipital cortices has been reported in some previous studies of stimulus-driven attentional orienting (Downar et al., 2000) and may reflect enhanced visual processing (Corbetta, 1998). To our knowledge, the present study is the first to provide evidence regarding the neural mechanisms involved in the impaired unconscious gaze-triggered attentional orienting in individuals with ASD.

The present results have several important implications. First, the results obtained under the subliminal condition show the neural mechanisms underpinning the impaired unconscious gaze-triggered attentional orienting in individuals with ASD (Sato et al., 2010), which may lead to a lack of joint attention in real life (Mundy et al., 1994). Previous behavioral studies on basic human perception have shown that humans consciously perceive only very restricted portions of the areas available for focused attention (Simons and Rensink, 2005). Certain brain regions, such as the amygdala, have supposedly evolved to monitor the environment without conscious awareness and to detect biologically significant stimuli (Vuilleumier, 2005). Together with these data, our results indicate that, when averted eyes are shown in areas lacking attentional resources, brain regions, such as the amygdala, are unconsciously stimulated to detect and activate the attentional system in TD individuals. In contrast, amygdala activation in response to social stimuli is generally weak in individuals with ASD; hence, it fails to detect eyes reflexively. Because many everyday life situations require humans to respond to eye-gaze signals that appear in a peripheral field or unconsciously, the deficit in unconscious gaze-triggered attentional orienting may account for the deficit in joint attention in individuals with ASD. These explanations suggest the possibility that behavioral impairments associated with gaze-triggered attentional orienting in individuals with ASD may be modified by treatments directed at amygdala activity. Possibly consistent with such an idea, preliminary evidence indicated that electrical stimulation of the amygdala in individuals with ASD modifies their autistic symptoms and can induce eye-contact communication (Sturm et al., 2013). Future research should examine the effect of such treatment on unconscious gaze-triggered attentional orienting in ASD individuals.

Second, the results from the supraliminal condition extend behavioral data showing comparable conscious gaze-triggered attentional orienting in TD and ASD individuals (e.g., Kylliäinen and Hietanen, 2004) and suggest that, similar to TD individuals, individuals with ASD can activate the attentional neural network in response to consciously viewed eye gaze. Thus, the psychological and neural mechanisms for reflexive joint attention may not be critically impaired in individuals with ASD when they can consciously view eye gaze in advance. These ideas are consistent with the results of behavioral intervention in which appropriate training drastically improved joint attention behaviors in individuals with ASD (MacDonald et al., 2014). The present data suggest that advanced instruction to pay sufficient attention to others’ eyes and to keep consciously perceiving them may facilitate typical reflexive joint attention behaviors in individuals with ASD.

Several limitations of the present study should be acknowledged. First, only high-functioning adults with mild ASD were tested, and, therefore, it remains unknown whether the current findings could be generalized to low-functioning adults, children, or more severe types of ASD. Given the previous findings showing comparable performance in TD and low-functioning ASD groups (Okada et al., 2003), individuals with low-functioning ASD may also show activation in the attentional network during conscious viewing of eye gaze. Second, although the present preliminary behavioral and fMRI analyses did not reveal significant effects of sex and age, sex was not balanced, and there was a narrow range of ages among the participants. Because previous studies have reported that these factors have modulatory effects on neural activation in individuals with ASD (e.g., Schneider et al., 2013; Joseph et al., 2015), it may be possible to identify the manner in which sex differences and developmental changes are associated with neural activity related to conscious and unconscious gaze-triggered attention orienting in individuals with ASD. Third, different paradigms were used to investigate the supraliminal and subliminal conditions (i.e., without and with mask images) and statistical comparisons of these presentation conditions were not conducted. Therefore, interpretations of the quantitative and statistical comparisons of conscious and unconscious gaze-triggered attention orienting in TD and ASD groups remain unclear. Finally, the fMRI interaction analysis of the subliminal data revealed small amygdala activation and broad (61.5% larger) activation outside the amygdala in the anterior temporal lobe cluster. This may be due, at least in part, to difficulties in accurately localizing amygdala activation. The amygdala is vulnerable to susceptibility-induced signal loss (Merboldt et al., 2001), which results in inaccurate overlap between functional and structural images after rigid body registration because they do not have the same brain shape. Methodological improvements in functional image acquisition, such as the use of small in-plane voxel size and small slice thickness (Olman et al., 2009) and optimal slice angle (Robinson et al., 2004) in combination with parallel imaging technique (Schmidt et al., 2005; Bellgowan et al., 2006), may more accurately localize amygdala activation in response to subliminally presented eyes. Future studies will be necessary to investigate these issues and to further elucidate the neural mechanisms underlying conscious and unconscious gaze-triggered attentional orienting in individuals with ASD.

In conclusion, the conjunction analysis of the fMRI data from the supraliminal condition revealed that the bilateral temporo–parietal regions and the right inferior frontal gyrus were activated in response to conscious averted eyes in both the ASD and TD groups. The interaction analysis under the subliminal condition showed that the left anterior temporal region, including the amygdala, was less activated in the ASD group compared with the TD group during attentional orienting triggered by unconscious eyes. These results indicate that these neural mechanisms underlie the impairments in unconscious but not conscious gaze-triggered attentional orienting in individuals with ASD.

## Author Contributions

WS, TK, SU and MT designed the research; obtained the data; WS, TK, SY and MT analyzed the data; and all authors wrote the manuscript. All authors read and approved the final manuscript.

## Conflict of Interest Statement

The authors declare that the research was conducted in the absence of any commercial or financial relationships that could be construed as a potential conflict of interest.

## Footnotes

^1^http://www.fil.ion.ucl.ac.uk/spm

^2^http://imaging.mrc-cbu.cam.ac.uk/imaging/BrodmannAreas

^3^http://www.mccauslandcenter.sc.edu/crnl/mricron/
